# Mitophagy Effects of Protodioscin on Human Osteosarcoma Cells by Inhibition of p38MAPK Targeting NIX/LC3 Axis

**DOI:** 10.3390/cells12030395

**Published:** 2023-01-21

**Authors:** Chien-Feng Huang, Yi-Hsien Hsieh, Shun-Fa Yang, Chao-Hung Kuo, Pei-Han Wang, Chung-Jung Liu, Renn-Chia Lin

**Affiliations:** 1 Department of Critical Care Medicine, Tungs’ Taichung MetroHarbor Hospital, Taichung City 435403, Taiwan; 2 Institute of Medicine, Chung Shan Medical University, Taichung City 40201, Taiwan; 3 Department of Medical Research, Chung Shan Medical University Hospital, Taichung City 40201, Taiwan; 4 Division of Gastroenterology, Department of Internal Medicine, Kaohsiung Medical University Hospital, Kaohsiung 80756, Taiwan; 5 Regenetative Medicine and Cell Therapy Research Center, Kaohsiung Medical University, Kaohsiung 80756, Taiwan; 6 Department of Medicine, Faculty of Medicine, College of Medicine, Kaohsiung Medical University, Kaohsiung 80756, Taiwan; 7 Department of Medicine, Kaohsiung Municipal Hsiao-Kang Hospital, Kaohsiung 80756, Taiwan; 8 Department of Orthopedics, Chung Shan Medical University Hospital, Taichung City 40201, Taiwan; 9 School of Medicine, Chung Shan Medical University, Taichung City 40201, Taiwan

**Keywords:** protodioscin, human osteosarcoma cell, apoptosis, p38MAPK, mitophagy, NIX, LC3

## Abstract

Protodioscin (PD) is a steroidal saponin with various pharmacological activities, including neuro-protective, anti-inflammatory, and anti-tumor activities. However, the effect of PD on human osteosarcoma (OS) cells is unclear. In this study, we found that PD significantly inhibits the growth of human HOS and 143B OS cells through the upregulation of apoptotic-related proteins (cleaved caspase-3, cleaved caspase-9, and cleaved PARP) and mitophagy-related proteins (LC3B and NIX), which contribute to the induction of apoptosis, and MMP (mitochondrial membrane potential) dysfunction and mitophagy. The inhibition of LC3 or NIX was shown to decrease apoptosis and mitophagy in PD-treated OS cells. The knockdown of p38MAPK by siRNA decreased mitochondrial dysfunction, autophagy, mitophagy, and the NIX/LC3B expression in the PD-treated OS cells. A binding affinity analysis revealed that the smaller the KD value (−7.6 Kcal/mol and −8.9 Kcal/mol, respectively), the greater the binding affinity in the PD-NIX and PD-LC3 complexes. These findings show the inhibitory effects of PD-induced mitophagy in human OS cells and may represent a novel therapeutic strategy for human OS, by targeting the NIX/LC3 pathways.

## 1. Introduction

Osteosarcoma (OS) is the most common primary malignant bone tumor in children, adolescents, and young adults, and accounts for approximately 20% of all primary bone cancers [1]. Complete radical resection is the typical treatment; however, approximately 80% of OSs may eventually develop a metastatic potential. A combination of advanced surgical techniques and new chemotherapies has improved the frequency of limb-sparing surgeries and the prognosis for OS patients, with an increase in the 5-year survival rate, from 20% to approximately 55–70% [2]. However, for patients with metastases, the 5-year survival rate remains at less than 20% [3]. As such, new strategies for targeting OS progression are needed to improve patient survival.

Autophagy is an intracellular degradation system that degrades the dysfunctional proteins and organelles in a lysosome-dependent manner [4]. A functional mitophagy has been shown to inhibit the accumulation of the damaged mitochondria, and then prevent carcinogenesis, and thus plays an important role in mitochondria quality control [5]. Some evidence suggests that the dysregulation of mitophagy contributes to the progression of some human diseases. Several proteins are mitophagy-targeting proteins, including BNIP3, PINK1/PARKIN, NIX, and FUNDC1, which are related to controlled hypoxia, cell death, mitochondrial depolarization, and cellular signaling pathways [6]. Mitophagy activated by the PINK/Parkin pathway removes the dysfunctional mitochondria and reduces the production of reactive oxygen species (ROS), thereby limiting the development of tumors by ROS [7]. Moreover, an enhanced mitophagy is beneficial for the treatment of cancers. For example, zinc oxide nanoparticles (NPs) can induce mitochondrial damage and ROS in oral squamous cell carcinoma (OSCC) cells, which causes apoptosis via excessive PINK1/Parkin-mediated mitophagy [8]. Unlike PINK1/Parkin-mediated mitophagy, BNIP3- and NIX (BNIP3L)-mediated mitophagy is independent of mitochondrial ubiquitination. The mitochondrial adaptors BNIP3 and NIX can directly interact with the mitochondria and target the autophagosomes [9]. The interaction of the NIX LIR motif with LC3 can promote mitophagy by recruiting autophagosomes to target the mitochondria, or by enhancing the formation of autophagosomes [5]. NIX plays a key role in tumorigenesis by mediating the Parkin translocation on the mitochondria and the subsequent PINK1/Parkin-mediated mitophagy [9]. In melanoma, NIX can recruit the orphan nuclear receptor TR3 to the mitochondria and subsequently cause melanoma cell death [10]. The aforementioned findings suggest that NIX can act as a tumor suppressor and affect tumor cell survival through the induction of mitophagy.

Protodioscin (PD) is a steroidal saponin that can be extracted from *Dioscorea nipponica* and *Tribulus terrestris*, and has been shown to improve glucose intolerance, metabolic syndrome, as well as renal injury [11] and anticancer effects [12,13]. PD has been shown to possess a neuroprotective ability against cerebral ischemia-reperfusion injury via intervention with inflammation and apoptosis [14]. PD can induce cell death in HL-60 leukemic cells through the progression of apoptosis [15], and has been shown to be cytotoxic against 60 human cancer cell lines [16].

However, the anticancer effects and the molecular mechanisms of PD against OS have not been investigated. In this study, we demonstrate that PD inhibits human OS cell growth by activating NIX/LC3 pathway-mediated mitophagy.

## 2. Materials and Methods

### 2.1. Chemicals and Reagents

Protodioscin (PD; CFN99517) with 99% purity was purchased from ChemFaces (Wuhan, Hubei, China). The MTT, DAPI (4′, 6-diamidino-2-phenylindole), and Hoechst 33258 were obtained from Sigma-Aldrich (St. Louis, MO, USA). Primary antibodies against cleaved caspase-9 (9508S) and cleaved PARP (9542S) were purchased from Cell Signaling Technology (Danvers, MA, USA). LC3 antibody (NB100-2220) was purchased from Novus Biologicals (Centennial, CO, USA). Antibodies against cleaved caspase-caspase-3 (SC-56053), PINK1 (SC-518052), Parkin (SC-32282), NIX (SC-166332), BNIP3 (SC-56167), total p38MAPK (SC-7972), phosphoryl p38MAPK (SC-166182), total Akt (SC-56878), phosphoryl Akt (SC-271966), LC3 siRNA (SC-43390), NIX siRNA (SC-37453), and horseradish peroxidase-conjugated secondary antibodies (anti-mouse and anti-rabbit) were purchased from Santa Cruz Biotechnology, Inc. (Santa Cruz, CA, USA). GAPDH (60004-1-Ig) antibody was purchased from Proteintech Group, Inc. (Rosemont, IL, USA). The p38 siRNA (MAPK14-Homo-760) was purchased from AllBio Company (Taichung, Taiwan).

### 2.2. Cell Lines and Culture Conditions

Two OS cell lines, HOS (BCRC Number: 60308) and 143B (BCRC Number: 60439) were obtained from the Bioresource Collection and Research Centre (BCRC), and one normal mouse osteoblast cell line, MC3T3-E1, was provided by Professor Chih-Hsin Tang (Department of Pharmacology, China Medical University, Taichung, Taiwan). The cell lines were maintained in minimum essential medium (MEM) with 10% (*v*/*v*) fetal bovine serum (FBS) and 1% penicillin-streptomycin (Invitrogen Life Technologies, Carlsbad, CA, USA) in a humidified atmosphere of 5% CO_2_ at 37 °C.

### 2.3. Cell Proliferation Assay

Cell proliferation was determined using an MTT assay. MC3T3-E1, HOS, and 143B cells (5 × 10^4^ cells/well) were seeded in 24-well plates (Greiner Bioone, Frickenhausen, Germany) and treated with PD (0, 2, 4, 6, 8, and 10 µM) for 24 or 48 h, and then incubated with MTT solution (0.5 mg/mL) for 4 h. Isopropanol was added to dissolve the crystal violet, and absorption at 570 nm was measured using a Multiskan MS ELISA reader (Labsystems, Helsinki, Finland).

### 2.4. Measurement of Apoptotic Profile and Mitochondrial Membrane Potential (MMP)

The apoptotic effect of PD treatment of OS cells was determined using a Muse^®^ Annexin V & Dead Cell Kit (Luminex Corporate, Austin, TX, USA). Briefly, OS cells (4 × 10^5^ cells/well) were seeded in 6-well plates in the presence of PD (0, 2, 4, and 6 µM) and cultured for 24 h. Cells were then trypsinised and Muse^®^ Annexin V & Dead Cell reagent was added. After 30 min, the PD-treated cells were collected and washed twice with ice cold PBS, and then the Muse^®^ MitoPotential Kit reagents (Luminex Corporate, Austin, TX, USA) were added and the cells were incubated for 20 min. Flow cytometry was performed using the Muse^®^ Cell Analyzer (Millipore, Hayward, CA, USA).

### 2.5. Measurement of Autophagy and Mitophagy

HOS and 143B OS cells treated with PD (1 × 10^4^ cells/well) were seeded on 8-well Lab-Tek Chambered Coverglass and incubated for 24 h. For the autophagy assay, cells were washed with PBS 3 times, 5 min each time, and then fixed with 4% paraformaldehyde for 10 min. Cells were treated with the Hoechst 33258 reagent (nucleus marker) at room temperature for 1 h, and then were stained using an AO reagent for 30 min. For the mitophagy assay, PD-treated cells were stained using a Dojindo Mitophagy Detection Kit (Dojindo EU GmbH, Munich, Germany) to detect lysosomes and the mitophagy effect. The cells were observed with a confocal microscope (Zeiss LSM 510 META, Heidelberg, Germany), and analyzed according to the manufacturer’s protocols.

### 2.6. siRNA Transient Transfection

The siRNA-LC3 (si-LC3, 10 nM), or siRNA-NIX (si-NIX, 10 nM), and RNAiMAX reagents (Thermo Fisher Scientific, Waltham, MA, USA) were mixed in serum-free MEM for 10 min, respectively. HOS cells were treated with the above complexes for 6 h, then the medium was changed to serum MEM and incubation was performed for 18 h. After 24 h, the cells were treated with PD (4 µM) for another 24 h, and then the total lysate was collected to measure the knockdown efficiency of si-LC3- or si-NIX-transfected cells.

### 2.7. Immunoblotting

Total protein was extracted from HOS and 143B cells with NETN buffer, and the total protein concentration was measured using a Bradford reagent (Bio-Rad, Hercules, CA, USA) and quantified with a spectrophotometer at 595 nm. Equal amounts of total protein (15 μg) from each cell line were subjected to 10% SDS-PAGE for protein separation for 70 min, and then transferred onto Immobilon^®^ PVDF membranes (MerckKgaA, Darmstadt, Germany) and blocked with nonfat dry milk (5%) for 1 h. The membranes were further incubated with the indicated primary antibodies (total caspase-3, cleaved caspase-3, total caspase-9, cleaved caspase-9, cleaved PARP, LC3B, PINK1, Parkin, NIX, BNIP3, total p38MAPK, phosphoryl p38MAPK, total Akt, phosphoryl Akt, and GAPDH) overnight, followed by incubation with secondary antibodies (anti-mouse or anti-rabbit IgG) for 1 h at room temperature. Specific antibody-bound protein bands were detected with an ECL Kit (Millipore, Bedford, MA, USA) using an ImageQuant LAS 4000 mini.

### 2.8. Molecular Docking

The AutoDock 4.2 software was used to determine the molecular interaction of PD with LC3 or NIX. The three-dimensional (3D) structure of LC3 (PDB ID: 5XAC; accessed on 12 July 2017) and NIX (ID: 4k6jA; accessed on 1 November 2022) was analyzed using the RCSB Protein Data Bank (https://www.rcsb.org/; accessed on 24 June 2020), ModBase (https://modbase.compbio.ucsf.edu/; accessed on 24 June 2020) and the chemical structure of PD was downloaded from PubChem (https://pubchem.ncbi.nlm.nih.gov/; accessed on 24 June 2005).

### 2.9. Statistical Analysis

Each experiment was repeated at least 3 times. Results were presented as the mean ± standard deviation, and statistical comparisons were made using the one-way analysis of variance (ANOVA) and Student’s *t* test. All analyses were performed using SPSS version 18.0 software. Statistical significance was defined as a value of *p* < 0.05.

## 3. Results

### 3.1. Effect of PD on Human OS Cell Viability

**Figure 1:**
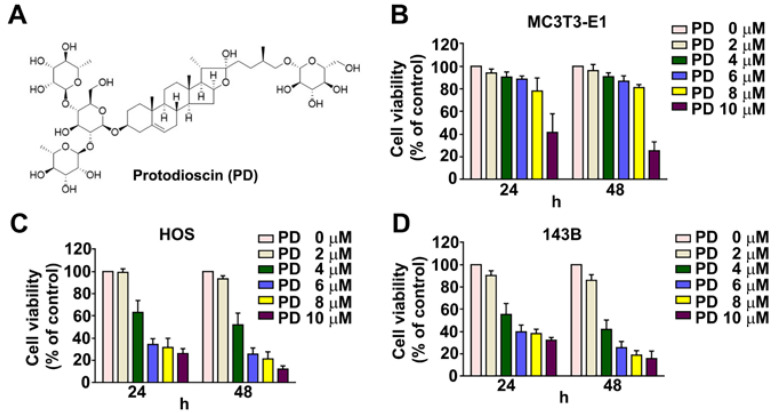
Effect of PD on the viability of human osteosarcoma (OS) cells. (**A**) Structure of protodi-oscin (PD). (**B**–**D**) MC3T3-E1 cells (normal osteoblast cells), HOS cells (human OS cells), and 143B cells (human OS cells) were exposed to various concentrations (0, 2, 4, 6, 8, and 10 μM) of PD for 24 h, and then the cell viability was measured using an MTT assay. ** *p* < 0.01 versus the control (0 μM, line 1). The results are the mean ± standard deviation of three experiments.

The structure of PD is shown in Figure 1A. The effect of PD on the viability of hu-man HOS and 143B OS cells was examined. HOS and 143B cells were treated with various concentrations (0, 2, 4, 6, 8, and 10 μM) of PD for 24 h and 48 h, and then analyzed using an MTT assay. As shown in Figure 1B, no significant effect on the cell viability of normal osteoblast cells (MC3T3-E1) treated with PD in concentrations at 2~6 µM for 24 h and 48 h, was observed. PD dose-dependently suppressed the cell viability in the HOS and 143B cells (Figure 1C, D). The IC50 values at 24 h and 48 h were 5.7 µM and 4.6 μM for the HOS cells, and the IC50 values were 5.1 µM and 3.8 µM for the 143B cell lines, respectively. These results indicate that a high concentration of PD (10 µM) has a cytotoxic effect on the growth of human OS cells and on normal osteoblast cells (MC3T3-E1). Therefore, we used PD concentrations of <8 µM in subsequent experiments.

### 3.2. Effect of PD on Apoptosis and Mitochondrial Dysfunction in Human OS Cells

**Figure 2:**
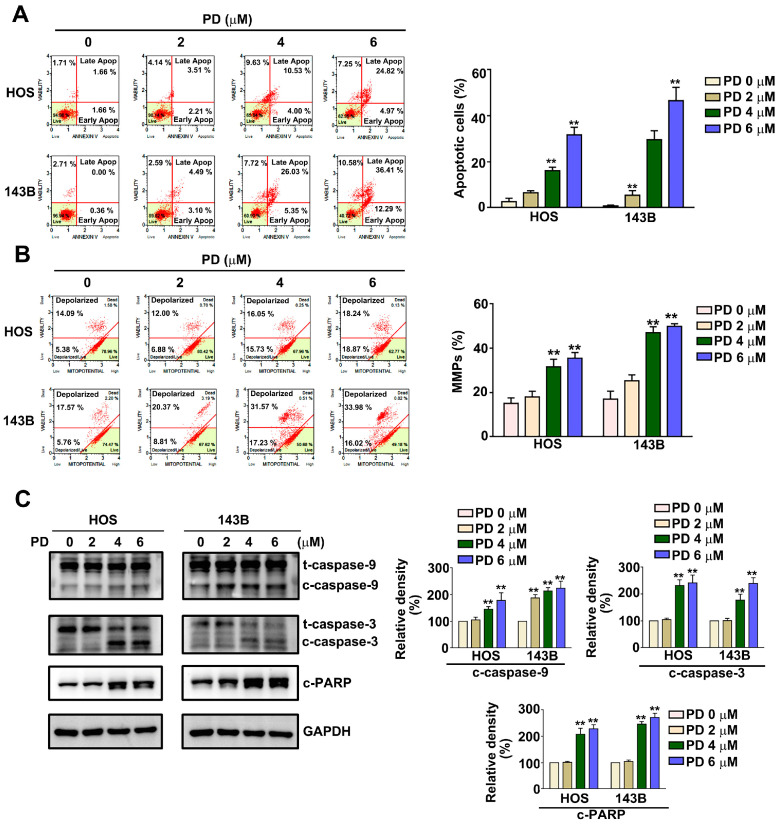
Effect of PD on apoptosis and MMP of human OS cells. Human HOS and 143B cells were treated with various concentrations (0, 2, 4, and 6 μM) of PD, and apoptosis and changes in MMP were detected. (**A**) Apoptotic cells and (**B**) MMP were detected with a Muse Cell Analyzer, an Annexin V assay, and a MitoPotential assay, respectively. (**C**) Apoptosis-related proteins (cleaved caspase-9, cleaved caspase-3, and cleaved PARP) in human HOS and 143B cells treated with PD (0, 2, 4, and 6 μM), were measured by immunoblotting. GAPDH was used as an internal loading control. ** *p* < 0.01, versus the control. The results are the mean ± standard deviation of three experiments.

To determine the effect of PD on apoptosis induction and mitochondrial membrane potential (MMP) changes in human HOS and 143B cells, the HOS and 143B cells were treated with various concentrations (0, 2, 4, and 6 µM) of PD for 24 h, and then the apoptotic profile and MMP changes were measured using an Annexin V/PI and JC-1 staining by Muse^®^ Cell Analyzer. The results show that the treatment with PD induced apoptosis from 3.23% to 29.79% in HOS cells and from 0.68% to 48.60% in 143B cells, respectively. (Figure 2A). We also found that PD induced the loss of mitochondrial membrane potential from 19.45% to 38.02% in HOS cells and from 23.23% to 50.21% in 143B cells, respectively (Figure 2B). An immunoblotting analysis indicated that the PD treatment resulted in the upregulation of apoptosis-related proteins (cleaved caspase-9, cleaved caspase-3, and cleaved PARP) in HOS and 143B cells (Figure 2C). These results indicate that PD induces apoptosis and MMP dysfunction in human OS cells.

### 3.3. Effect of PD on the Autophagy in Human OS Cells

**Figure 3:**
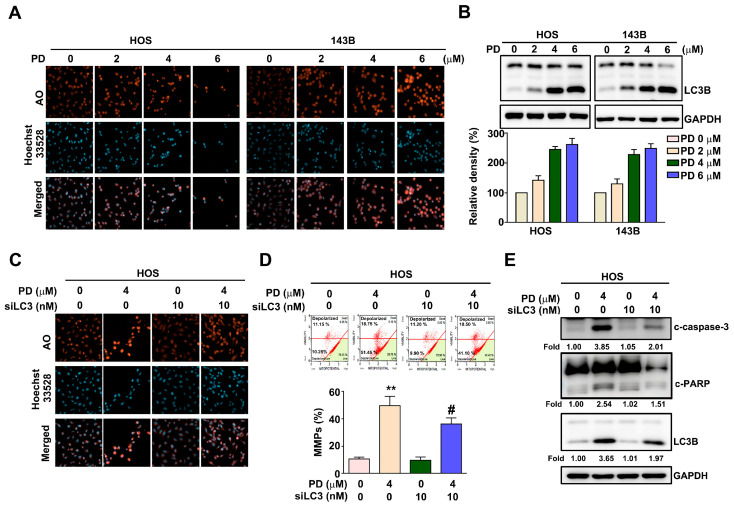
Effect of PD on the autophagy and apoptosis in human OS cells. (**A**,**B**) Human HOS and 143B OS cells were treated with various concentrations (0, 2, 4, and 6 μM) of PD, and then the autophagy effect by AO staining was examined and the marker of autophagy, LC3B, was measured. (**C**–**E**) LC3 siRNA reversed PD-induced autophagy (AO staining), MMP changes, and apoptosis. Apoptotic cleaved caspase-3, cleaved PARP proteins, and the autophagy marker LC3B were measured in HOS cells exposed to PD (4 μM), and/or LC3 siRNA (10 nM), and then were measured by immunoblotting. GAPDH was used as an internal loading control. ** *p* < 0.01, versus the control. # *p* < 0.05 versus the PD-only treatment (line 2). The results are the mean ± standard deviation of three experiments. Scale bar: 50 μm.

The effect of PD on the induction of autophagy in human OS cells was examined using Acridine Orange (AO) staining to assess the volume of acidic vesicular organelles (AVO), which is autophagy induction. HOS and 143B cells were treated with various concentrations of PD (0, 2, 4, and 6 µM) for 24 h. The results show that PD induced autophagy by AO staining (Figure 3A), and in the significantly upregulated autophagy marker LC3B in both HOS and 143B cells, in a dose-dependent manner (Figure 3B). LC3 is crucial for the induction of cell autophagy. To investigate the role of LC3 in PD-induced autophagy, human HOS cells were treated with PD (4 µM) for 24 h in the presence of LC3 siRNA (10 nM), to demonstrate that LC3 knockdown reduced PD-induced autophagy (Figure 3C), MMP changes (Figure 3D), and the protein expression of cleaved caspase-3, cleaved PARP, and LC3B in HOS cells (Figure 3E). These results suggest that PD induces autophagy-dependent apoptosis in human OS cells.

### 3.4. Effect of PD on the Mitophagy in Human OS Cells

**Figure 4:**
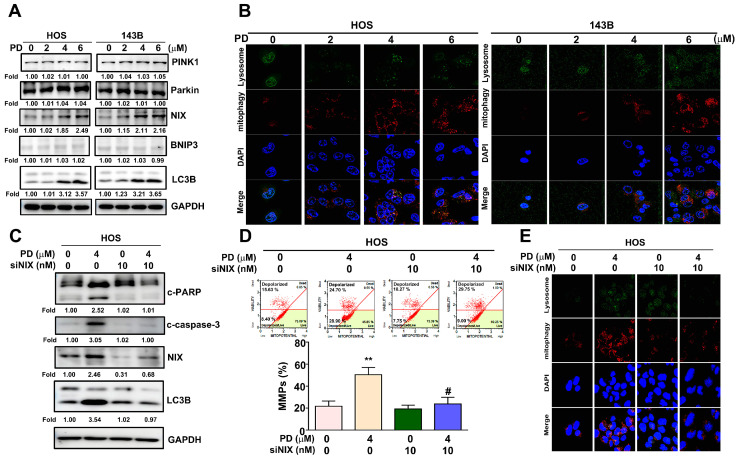
Effect of PD on mitophagy in human OS cells. Human HOS and 143B cells were treated with various concentrations (0, 2, 4, and 6 μM) of PD. (**A**) The expression of mitophagy-related proteins was detected with immunoblotting. (**B**) Induction of mitophagy using an immunofluorescence assay with a mitophagy detection kit. (**C**) The expressions of cleaved caspase-3, cleaved PARP, NIX, and LC3B were measured using immunoblotting assays, (**D**) and MMP changes and (**E**) mitophagy induction in the human HOS cells were exposed to PD (4 μM) and/or NIX siRNA (10 nM). GAPDH was used as an internal loading control. ** *p* < 0.01, versus the control. # *p* < 0.05 versus the PD-only treatment (line 2). The results are the mean ± standard deviation of three experiments. Scale bar: 50 μm.

To further identify the effect of PD on the induction of mitophagy and mitophagy-related apoptosis in human OS cells, different PD concentrations (0, 2, 4, and 6 µM) were detected in the HOS and 143B OS cells that were treated for 24 h. Subsequently, mitophagy detection, MMP measurements, and immunoblotting assays were performed. As shown in Figure 4A, PD significantly upregulated NIX and LC3B in the HOS and 143B cells in a dose-dependent manner, and no obvious changes were observed in the expression levels of PINK1, Parkin, and BNIP3. The damaged mitochondria fused to a lysosome and then Mtphagy Dye emitted a high fluorescence in the mitophagy induction. Our results observed that the fluorescent intensity of Mtphagy Dye was increased in the PD-treated OS cells dosed with 4 and 6 µM PD, compared with the control cells (Figure 4B). Some evidence has revealed that NIX interacts with LC3 to form a mitochondria-NIX-LC3-autophagosome complex in mitophagy progression [17]. To explore the role of NIX in the PD-induced mitophagy in human HOS cells, the cells were treated with PD (4 µM) for 24 h in the presence of NIX siRNA (10 nM). We found that NIX siRNA reduced the PD-upregulated level of the apoptotic-related proteins (cleaved PARP, cleaved caspase-3), mitophagy-related proteins (NIX and LC3B), MMP changes, and mitophagy induction (Figure 4C–E).

### 3.5. p38 MAPK Mediates PD-Induced Mitophagy in Human OS Cells

**Figure 5:**
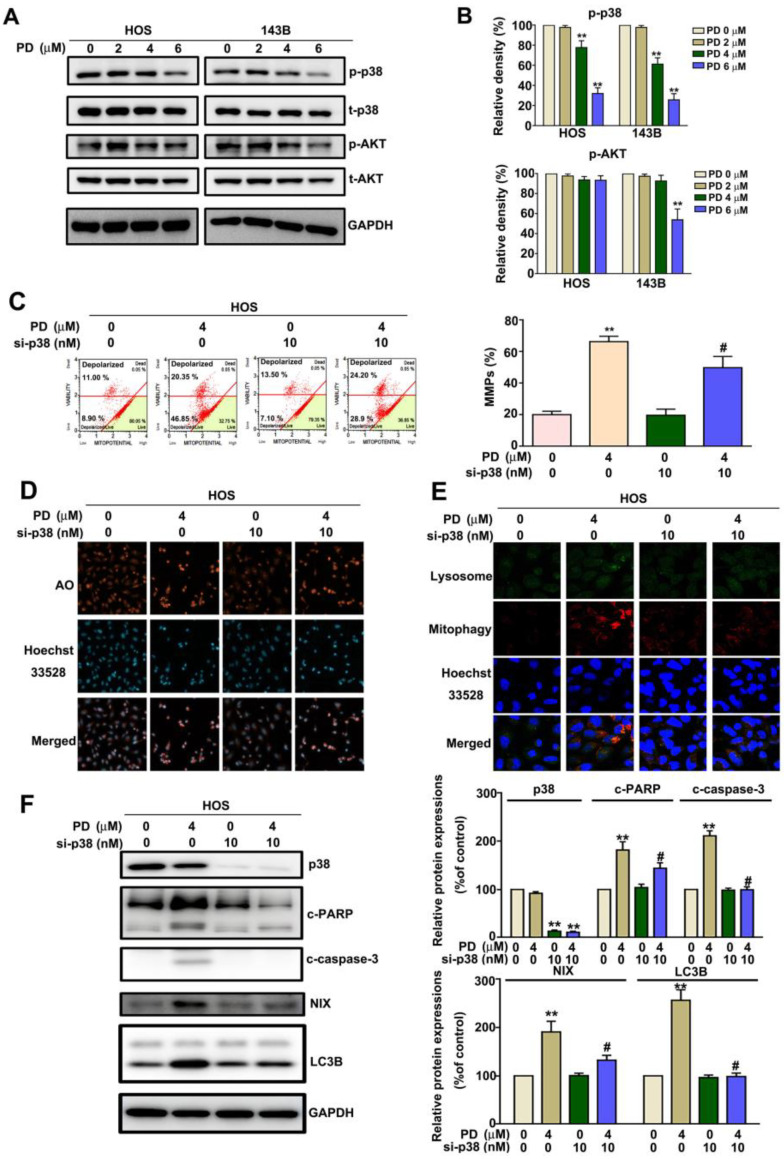
The p38MAPK signaling pathway mediates the PD-induced mitophagy and apoptosis in human OS cells. (**A**,**B**) Human HOS and 143B OS cells were exposed to various concentrations (0, 2, 4, and 6 μM) of PD for 30 min. Cells were harvested and examined with immunoblotting to deter-mine the signaling pathway activation. HOS cells exposed to 4 μM PD and/or p38 siRNA (10 nM) were harvested for detecting (**C**) MMP changes, (**D**) AO staining, (**E**) mitophagy induction, and (**F**) the protein expressions of p38, cleaved caspase-3, cleaved PARP, NIX, and LC3B proteins, using immunoblotting assays. GAPDH was used as an internal loading control. ** *p* < 0.01, versus the control. # *p* < 0.05 versus the PD-only treatment (line 2). The results are the mean ± standard deviation of three experiments. Scale bar: 50 μm.

To investigate which signal transduction pathway is involved in the PD-induced mitophagy, we treated the HOS and 143B cells with PD (0, 2, 4, and 6 µM). The cells were then harvested and examined using immunoblotting, to observe the phosphorylation/activation of the signaling pathways. We found that PD significantly reduced the phosphorylation/activation of the p38MAPK signaling pathway (Figure 5A,B). The role of p38MAPK in the PD-induced mitophagy progression was further confirmed by treating the HOS cells with p38 siRNA. The results revealed that p38 siRNA reduced the PD-induced MMP changes (Figure 5C), autophagy induction by AO staining (Figure 5D), mitophagy induction (Figure 5E), and the apoptotic-related proteins (cleaved PARP and cleaved caspase-3), and mitophagy-related proteins (NIX and LC3B) (Figure 5F). These results demonstrate that PD induces the NIX-mediated mitophagy by inhibiting the p38MAPK activation in human HOS cells.

### 3.6. PD-NIX and PD-LC3 Complexes Provide the PD-Regulated Mitophagy

**Figure 6:**
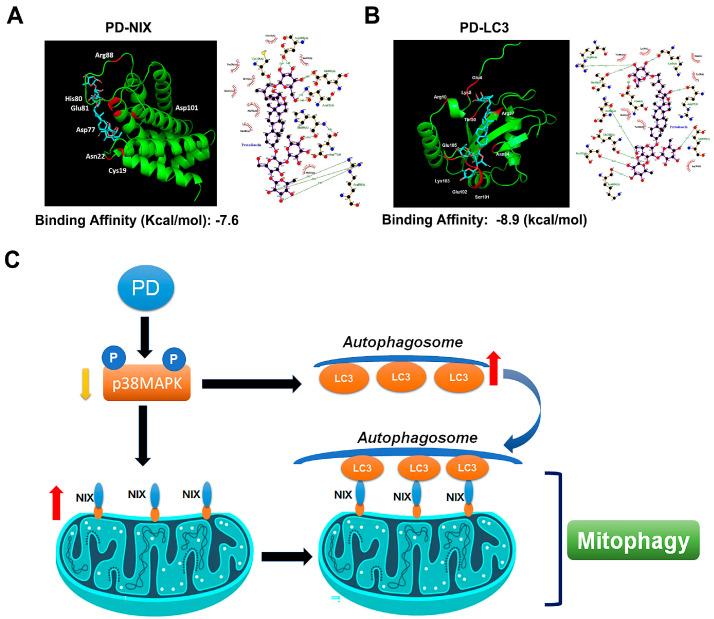
Three-dimensional surface representation of the interactions of the PD-NIX/PD-LC3 complex. (**A**) Three-dimensional ribbon diagrams representing the PD-NIX complex and the (**B**) PD-LC3 complex at the level of the residues. (**C**) Illustration of our proposed mechanism of the PD-induced mitophagy, by targeting the NIX/LC3 pathways in human OS cells.

To characterize the binding affinity between PD and NIX, and between PD and LC3, we used isothermal titration calorimetry (ITC). These results indicated that the smaller the KD value (−7.6 Kcal/mol and −8.9 Kcal/mol, respectively), the greater the binding affinity in the PD-NIX and PD-LC3 complexes. Crystallization screening was performed using the crystal of a chimeric construction containing the PD-NIX or PD-LC3 complex. The structures were resolved to show the interactions between PD and NIX (Figure 6A), and between PD and LC3 (Figure 6B). The results indicate that PD inhibits tumor growth by targeting the NIX- and LC3-mediated mitophagy in human OS cells (Figure 6C).

## 4. Discussion

Despite the development of new drugs for the treatment of OS, the survival rate of OS patients remains low. Thus, the development of novel approaches and anticancer treatments for OS are needed. PD is a steroidal saponin found in *Dioscorea nipponica* and *Tribulus terrestris*, which are both traditional herbs. There are a number of novel results in this current study: (1) PD significantly decreased the growth of human OS cells in a dose-dependent manner; (2) PD significantly induced cell apoptosis, MMP changes, autophagy, and mitophagy through the upregulation of the apoptotic cleaved caspase-9, cleaved caspase-3, cleaved PARP, and mitophagic LC3/NIX protein expression; (3) the LC3 or NIX knockdown reduced the PD-induced MMP changes, autophagy, and apoptosis through the downregulation of LC3B, cleaved caspase-3, and cleaved-PARP; (4) p38MAPK was involved in PD-induced MMP changes, autophagy, and mitophagy induction; and (5) PD inhibited the OS cell growth through the formation of the PD-NIX and PD-LC3 complexes, thereby inducing the autophagosome formation and mitophagy induction (Figure 6C).

Mitophagy (mitochondrial autophagy) plays a critical role in regulating carcinogenesis and the cancer progression by acting as a tumor promoter or suppressor. Mitophagy, induced by guangsangon E (GSE), results in a mitochondrial dysfunction, including a membrane potential loss, mitochondria fission, and ROS accumulation, which finally leads to cell death in vitro, and significantly reduces the size and weight of the MDA-MB-231 tumors, as shown in a xenograft murine tumor model [18]. The activation of the PINK1/Parkin-mediated mitophagy by aloe gel glucomannan (AGP), significantly induces the cell death of colon cancer cells through the accumulation of impaired and ROS-generating mitochondria [19]. Apoptin causes the loss of MMP and increases ROS-mediated mitophagy through an increase in NIX expression in human liver cancer cells [20]. In addition, puerarin has been shown to alleviate the Cd-induced mitochondrial dysfunction and the inhibited PINK1-Parkin and Nix-regulated mitophagy in rat cortical neurons in vitro and in vivo [21]. Methyl protodioscin (MPD) and protodioscin (PD) are bioactive steroidal saponins from the rhizoma of Polygonatum sibiricum. Accumulated evidence places a major focus on the protective effect of methyl protodioscin (MPD) against tumorogenesis and apoptosis induction in various tumor cells, such as pancreatic cancer cells [22], osteosarcoma cells [23], oral squamous cell carcinoma [24], and lung cancer cells [25]; however, there is less experimental evidence to clarify the antitumor effect and the related molecular mechanisms of PD in various tumor cells. For example, PD induces apoptosis through ROS-mediated ER stress via the JNK/p38 activation pathway in human cervical cancer cells [13]. Similar apoptotic effects of PD have been suggested in human bladder cancer cells and liver cancer cells [12,26]. Only one study found that the frostane-type steroidal saponin protodioscin, from the rhizome of Dioscorea tokoro, induced cell apoptosis and autophagy, which resulted in p62 and LC3-II accumulation in HepG2 cells [27]. The findings from the earlier study indicate that mitophagy induction by PD causes the death of human OS cells through the NIX-LC3 complex formation.

The p38MAPK plays an important role in cancer progression, such as in-cell proliferation, cell metastasis, cell death, and mitophagy; p38 has been shown to promote breast cancer cell proliferation and lung metastasis [28]. Some natural compounds that regulate tumor progression are involved in the p38MAPK signaling pathways, such as EGCG that inhibit OC differentiation by regulating mitophagy-mediated AKT/p38MAPK pathways [29]. p38MAPK regulates licochalcone A (LicA)-executed antitumor activity against human OS cells through the modification of mitochondria-mediated intrinsic apoptotic pathways in vitro and in vivo [30]. Based on these observations, p38MAPK has an essential role in mitophagy induction in PD-treated OS cells. A recent study showed that p38MAPK plays an important role in the regulation of mitophagy in tumor cells. Dr. Ko et al. reported that the activation of p38MAPK mediates sustained adenosine-induced mitochondrial ROS production, which then promotes mitochondrial fission and mitophagy [31]. The activation of p38 mediates Eclalbasaponin I (EcI)-regulated mitophagy and apoptosis in human SK-N-SH neuroblastoma cells [32]. P38MAPK has also been reported to mediate oleanolic acid (OA)-induced mitophagy and apoptosis, and chemosensitization to 5-fluorouracil (5-FU) in human colon cancer cells [33]. The manipulation of mitophagy by an “all-in-one” nanosensitizer, augments sonodynamic glioma therapy through p38MAPK-PINK1-PRKN-dependent mitophagy and apoptosis [34]. Accordingly, we have first demonstrated that the high levels of NIX/LC3B expression significantly induced mitophagy and apoptosis by p38MAPK activation in human OS cells. These studies are expected to suggest that PD possesses anti-tumor effects in vitro, and thus further research is needed to investigate the antitumor and mitophagy effects in in vivo animal models. Therefore, PD could be considered a potential antitumor agent against human OS. Recently, clinical studies have demonstrated the safety assessment and efficiency of PD for human health. Furosap (FS) with 20% protodioscin (PD)-enriched extract (FurosapTM) was examined in 50 male volunteers to observe the efficacy of FS (500 mg/day). These clinical trial results found that volunteers taking FurosapTM demonstrate obvious improvements in mental alertness, mood, cardiovascular health, and libido. There were no observed influences to volunteers’ clinical blood parameters, such as cholesterol, triglyceride (TG), nephrotoxicity, hepatotoxicity, high-density lipoprotein (HDL) and low-density lipoprotein (LDL) levels [35]. Therefore, FurosapTM (enriched in 20% protodioscin) is safe and effective for human use.

## 5. Conclusions

We conclude that PD induces apoptosis and mitophagy to block the p38MAPK activation by the NIX/LC3 axis in human OS cells. This might be the underlying mechanism whereby PD suppresses the progression of human osteosarcoma.

## Data Availability

The authors will freely release all data supporting the published paper upon direct request to the corresponding author.
